# The chain-mediating effects of negative physical sensation and experiential avoidance on exercise anxiety in college students

**DOI:** 10.3389/fpsyg.2024.1465424

**Published:** 2024-11-26

**Authors:** Yi Wang, Jing Tian, Qingxuan Yang

**Affiliations:** ^1^School of Physical Education, Weinan Normal University, Weinan, China; ^2^School of Foreign Languages Studies, Weinan Normal University, Weinan, China; ^3^Department of Physical Education, Chang'an University, Xi'an, China

**Keywords:** endurance exercise, exercise anxiety, negative physical sensation, experiential avoidance, mediating effects

## Abstract

**Purpose:**

The present study aimed to explore the potential mediating role of negative physical sensation and experiential avoidance in the association between endurance exercise and exercise anxiety among university students.

**Method:**

In this study, a questionnaire method was employed to conduct the Adolescent Athlete Non-Intellectual Factors Survey Scale on 1,200 college students. From this sample, 287 individuals with exercise anxiety were identified through an endurance exercise test and the Acceptance and Action Questionnaire (AAQ-II) subsequently administered as well as The Borg Rating of Perceived Exertion (RPE). Subsequently, statistical analyses including correlation, regression, and mediation were performed using SPSS26 as the analytical tool. Additionally, the bias-corrected nonparametric percentile Bootstrap method was used to test for the mediating effects and estimate the confidence intervals with 5,000 iterations, and the confidence interval (CI) was set at 95%. Finally, in AMOS24, a mediating construct was established by incorporating exercise anxiety as the dependent variable, endurance exercise behavior as the independent variable, and negative physical sensations and experiential avoidance as the mediating variables (*R* = 0.619, *R*^2^ = 0.384). A path analytic procedure was employed to test the hypotheses while percentile bootstrap analysis was conducted to examine the indirect effects.

**Results:**

The results show that endurance exercise negatively predicts negative physical sensations (*β* = −0.48, *p* < 0.001), negative physical sensation positively predicts experiential avoidance (*β* = 0.36, *p* < 0.001) and exercise anxiety (*β* = 0.40, *p* < 0.001), and experiential avoidance positively predicts exercise anxiety (*β* = 0.26, *p* < 0.001).

**Conclusion:**

There were significant correlations among endurance exercise, negative physical sensation, experiential avoidance and exercise anxiety. Endurance exercise affects exercise anxiety through two ways: (1) endurance exercise → negative physical sensation → exercise anxiety; (2) endurance exercise → negative physical sensation → experiential avoidance → exercise anxiety. Negative physical sensations and experiential avoidance play mediating and chain-mediating roles between endurance exercise and exercise anxiety.

## Highlights

- Negative physical sensation and experiential avoidance, as negative cognitive and emotional regulation methods, play an important role in behavior orientation and emotional disorders. These factors may elucidate the underlying mechanisms contributing to the onset and perpetuation of exercise anxiety.
- Endurance exercise behaviors can regulate exercise anxiety through the way of physical sensation and emotion strategies, and negative physical sensation and experiential avoidance play a chain mediating role on exercise anxiety in endurance exercise behavior.
- For sedentary individuals concerned about participating in endurance sports, it’s important to reduce their negative physical sensations during these activities, which can help alleviate their exercise anxiety and increase their participation in endurance exercise.
- The interventions of exercise anxiety can reshape physical cognition and emotional experience by participating in exercise, eliminating negative beliefs and avoidance, promoting positive sensations and emotions through exercise behavior, ultimately reducing exercise anxiety.

## 1 Introduction

Although an increasing number of studies suggest that physical activity has a beneficial intervention effect on mental disorders such as anxiety and depression (de Moor et al., 2006; Lafer et al., 2023), applying these findings to promote mental health for patients with anxiety depression-related disorders and others can be challenging (Mason et al., 2019), because individuals with high levels of anxiety-related traits (e.g., anxiety sensitivity) tend to report low levels of physical activity (Trigueros et al., 2022). Similarly, depression may result in reduced levels of physical activity (Endrigue et al., 2023). Brendon et al. conducted a cross-sectional study employing multivariable logistic regression analysis on community-based data from 47 countries worldwide, revealing a significant positive correlation between low levels of physical activity and anxiety (Stubbs et al., 2017). Consequently, despite the demonstrated benefits of physical activity/exercise in alleviating symptoms of depression and anxiety, a substantial proportion of individuals with mood and/or anxiety disorders do not engage in regular exercise (Pelletier et al., 2017; Liu and Cao, 2022). Depressed individuals exhibited a lower participation rate in physical activity compared to those non-depressed ones (Dai et al., 2019).

People with anxiety tend to do less physical activity, because the physical sensations caused by physical activities such as sweating, muscle tension and increased heart rate, are considered to be consistent with the physiological sensations of anxiety (Mckay et al., 2010). For people with low physical activity and sedentary behavior, exercises, especially the endurance exercises, may make them more anxious however (Weinstein et al., 2015). Exercise can lead to depression and anxiety, because the physical sensations generated by physical activity may stimulate anxiety, causing them to fear physical activity as the result. Therefore, exercise is not only a potent anxiolytic, but also a source of anxiety (Mason et al., 2019). Exercise anxiety as a stress response is usually caused by an individual’s assessment of his own exercise capacity in a specific sports situation. Therefore, exercise anxiety is a trait or state response of individuals to sports stress situations. It is a series of cognitive assessment, physical arousal and behavioral responses caused by the individual’s perception of potential stress (Ford et al., 2017). It is considered to be a mental reaction aroused by physical activity participation, testing and competition situations, often accompanied by unpleasant emotional experience of the exercise, which greatly affects participants’ performance on-the-spot and their physical activity participation in endurance exercise (Wang, 2004).

Anxiety sensitivity is a major risk factor for the development of anxiety symptoms among both adolescents and adults (Mclaughlin and Hatzenbuehler, 2009). It is reported that people with high anxiety and high anxiety sensitivity have lower levels of physical activity and exercise. There is a significant correlation between anxiety sensitivity and exercise frequency (Goodwin, 2003; Lanoye et al., 2022). The change in anxiety sensitivity precedes the change in depression caused by exercise. Changes in anxiety sensitivity mediate the beneficial effects of exercise on anxiety and depression (Smits et al., 2008). Therefore, exercise may affect emotional disorders by affecting anxiety sensitivity (Broman-Fulks et al., 2018). Individual’s beliefs in physically, psychologically and socially harmful consequences constitute the individual’s sensitivity to anxiety (Taylor, 1999). And, three lower-order factors measuring as physical concerns, psychological disorder concerns and social concerns constitute the secondary factor structure of anxiety sensitivity (Rodriguez et al., 2004). Individuals’ concerns about physical dimensions are the key factors affecting panic and post-traumatic stress disorder (Zinbarg et al., 1997). Therefore, anxiety sensitivity in physical dimension can prospectively predict the subsequent development of anxiety symptoms and panic attacks (Boffa et al., 2016). It has also been proven that emotions affected by physical sensation can predict the development and maintenance of anxiety in non-clinical samples (Mcwilliams and Asmundson, 2001; Stubbs et al., 2017). For sedentary people with low physical activity, physical activity, especially endurance exercise, maybe impact their exercise anxiety through the physical sensation of lower-order factors in anxiety sensitivity.

Therefore, we can surmise that the negative physical sensation (i.e., negative reactions to the physical sensations with exercise) is individual’s poor perception of one’s own physical health and exercise capacity, which may cause some psychological changes such as tension and anxiety. It is affected by potentially stressful situations, negative emotions and avoidance coping strategies, and has an important influence on individual emotion and behavior. Negative physical sensation refers to the perception and attitude toward one’s own physical health and exercise capacity under a specific stressful situation. It is caused by the emotional disorder under a stressful environment, which is the physical expression of the emotional disorder induced by the stressful environment. Negative physical sensation is closely related to emotional regulation. Avoidance coping strategies caused by negative physical sensation will reduce behavioral expression and lead to the increase of autonomic nerve activities such as respiration, heart rate, and skin electrical activity (Gross, 2002). Meanwhile, emotional regulation can change the negative physical sensation such as pain (Wang et al., 2016). Hence, negative evaluations of physical sensations and employment of avoidance coping strategies are strongly associated with heightened levels of tension and anxiety (Shin and Kemps, 2020).

In the stress situations, the avoidance strategy of cognition, emotion and behavior caused by negative evaluation of physical sensation and emotional disorder is called experiential avoidance. “Experiential avoidance refers to the tendency to engage in behaviors that function to avoid or escape from unwanted internal experiences, including thoughts, emotions, physical sensations and memories.” It is avoidance strategies and measures in cognition, emotions, and behavior, taken by individuals to deal with past negative memories in the context of stress (Hayes et al., 1996). As a self-protection mechanism, experiential avoidance plays an important role in behavioral and mental health. However, the excessive experiential avoidance is believed to produce and maintain the development of psychopathology (Chawla and Ostafin, 2007). Therefore, experiential avoidance has a mediating effect on a variety of psychological disorders. For example, experiential avoidance and emotional disorder function as the mediating variables, subsequently cause changes in sensitivity of behavioral inhibition and post-traumatic stress disorder. Experiential avoidance, as a negative regulation strategy for emotions, constitutes a risk factor for psychological and behavioral disorders (Maack et al., 2012; Warnke et al., 2018).

Some studies indicated that the experiential avoidance mediates the relationship between anxiety sensitivity and eating disorders (Espel-Huynh et al., 2019). In addition, experiential avoidance mediates the relationship between attachment anxiety and the frequency of paranoid idea. Experiential avoidance and difficulties engaging in goal-directed behavior mediate the relationship between the fear of cognitive decontrol, and publicly observable anxiety reactions and severity of depression symptoms. Experiential avoidance is more closely related to the development of severe depressive symptoms. Akbari examined four anxiety prediction models, and believed that experiential avoidance as an intermediary variable links different anxiety factors together and its explanation for anxiety of outcome variables is as high as 74%, and proposed the acceptance of internal experience as the theoretical basis for psychotherapy for generalized anxiety disorder (Akbari and Khanipour, 2018). Theories focusing on cognitive and motivational factors emphasize that individuals with anxious, depression tend to avoid behaviors (e.g., PA). This tendency limits positive experiences, reinforces inactivity, and contributes to the onset and maintenance of depression, that in turn prompts even more avoidance and thereby inactivity (Ottenbreit et al., 2014).

There is a close relationship between anxiety and behavioral inhibition guided by physical negative sensation and experience avoidance, and behavioral inhibition is the strongest predictor of anxiety. Therefore, negative physical sensation and experience avoidance, as negative cognitive and emotional regulation methods, play an important role in behavior orientation and emotional disorders. In addition, experiential avoidance mediates the relationship between attachment anxiety and the frequency of paranoid ideas (Castilho et al., 2017); experiential avoidance and difficulties engaging in goal-directed behavior mediate the relationship between the fear of cognitive decontrol, and fear of publicly observable anxiety reactions and the severity of depression symptoms (Ghazanfari et al., 2018). Experiential avoidance is more closely related to the development of severe depressive symptoms (Suzuki et al., 2018).

In spite of the current proliferation of research on promoting individuals’ mental well-being through sport and physical activity, yielding favorable outcomes, limited attention has been given to psychological interventions to enhance sport participation and performance among individuals with movement disorders. And the existing studies have not explored the underlying factors, such as mechanisms of impact and processes of participants’ anxiety about physical activity, especially forced endurance exercise. Consequently, it is crucial to investigate the mediating variables and influencing factors of exercise-induced anxiety in order to enhance engagement in physical activity among individuals with anxiety and depression, while simultaneously improving participants’ endurance performance. Results of the research can subsequently be utilized for intervening in psychological disorders among patients suffering from anxiety and depression. Therefore, it is imperative to investigate the factors, mechanisms, and maintenance of anxiety in physical activities, particularly endurance sports, among sedentary or exercise-anxious individuals. This research is crucial for health promotion and implementing exercise behavioral interventions, targeting individuals with anxiety and other mental health disorders. Furthermore, it holds significance in developing interventions to enhance their endurance performance.

Taken together, it is hypothesized that negative physical sensation and experiential avoidance play an important role as mediators in endurance exercise behaviors and exercise anxiety. To test this hypothesis, we investigate the association between exercise anxiety and endurance exercise behaviors, as well as negative physical sensations and experiential avoidance, among non-professionally trained college students. The purpose of this research is to construct an exercise anxiety model to illustrate the impact factors and mechanisms of participants’ exercise anxiety about physical activity, especially endurance exercise, to verify negative physical sensation as a mediating variables between endurance exercise behaviors and exercise anxiety.

## 2 Study tools and procedures

### 2.1 Participants

This study employs the cluster sampling method to select a sample of 1,200 students from two undergraduate colleges and Self-Reported Questionnaire of Non-Intelligent Factors for Young Athletes questionnaire test to test the anxiety tendency of these research participants.

Based on the results of a large-scale survey of Chinese adolescents conducted by Chinese scholar Zeng, it was shown that in “Self-Reported Questionnaire of Non-Intelligent Factors for Young Athletes questionnaire test,” the mean value of the sports anxiety dimension scores of young athletes with physical fitness as their dominant sport is (14.34 ± 2.83) (Jianchuan and Meijuan, 2012). Taking into account that the study population is ordinary university students without any training background, we set the inclusion criteria for the sample with a tendency to exercise anxiety as the mean value plus one criterion (14.34 + 2.83). All participants engaged in endurance exercise must meet the condition of a heart rate surpassing 135 beats per minute in during exercise and an exercise duration exceeding 10 min per session less than once a week. A total of 287 freshmen and sophomores were included, 192 females (66.9%) and 95 males (33.1%), with a mean age of 19.29.

### 2.2 Procedures

Differences in endurance running performance can be elucidated by three key factors: maximal oxygen uptake (VO2max), lactate threshold intensity, and running economy. In individuals previously untrained, aerobic high-intensity interval training (2–4 min at ≤100% of the speed associated with maximal oxygen consumption [VO2max]) enhances maximal oxygen uptake (VO2max) and augments endurance exercise performance. Conversely, for runners with training experience, higher-intensity interval training is imperative to attain the desired outcome (Gliemann et al., 2015). In terms of energy production, longer distance running exhibits a comparatively lower rate of energy production than shorter distances; for instance, the oxygen uptake cost for an 800 m run would be 95.4 rnl/kg/min, which significantly surpasses the maximal oxygen uptake capacity of a middle-distance runner. Consequently, middle-distance runs spanning from 800 to 3,000 m and taking approximately 2–10 min to complete rely on both aerobic and anaerobic energy systems (Brandon, 1995). The 800-m run has been demonstrated to primarily rely on aerobic capacity, specifically maximal oxygen uptake (VO2max), while approximately 40% of energy metabolism is contingent upon anaerobic energy metabolism (Craig and Morgan, 1998). Hence, engaging in middle to long distance running events such as 800 m and 1,000 m can effectively stimulate both the aerobic and anaerobic energy systems.

Therefore, middle to long-distance running, such as 800 m and 1,000 m, is efficacious in enhancing aerobic and anaerobic energy systems including maximal oxygen uptake (VO2max) and lactate threshold strength. These training modalities help improve cardiorespiratory fitness and lactate metabolism, and are extensively employed in endurance training and testing. In China, middle- and long-distance running (800 m for women and 1,000 m for men) is an important item in the national assessment of students’ physical fitness and health. However, individuals lacking a training background often experience heightened psychological stress due to physiological reactions such as physical fatigue, respiratory difficulties, and muscle soreness when engaging in 800 m and 1,000 m running.

All participants, who had previously completed a survey on exercise participation behaviors, completed the Self-Reported Questionnaire of Non-Intelligent Factors for Young Athletes, Acceptance and Action Questionnaire (AAQ-II), and The Borg Rating of Perceived Exertion (RPE) questionnaires 10 min prior to undergoing an endurance test (1,000 m for males and 800 m for females). The project was approved by the ethics academic committee, and all participants were given written informed consent.

### 2.3 Acceptance and action questionnaire (AAQ-II)

In 2004, based on Relational Frame Theory, Hayes compiled an Acceptance and Action Questionnaire-AAQ that incorporates 16 questions to measure experiential avoidance and psychological inflexibility (Hayes et al., 2004). Later, in 2011, the second edition of the Acceptance and Action Questionnaire (AAQ-II) was formed by Bond and Hayes, “the mean alpha coefficient is 0.84 (0.78–0.88), and the 3- and 12-month test–retest reliability is 0.81 and 0.79, respectively” (Bond et al., 2011). AAQ-II is becoming a measuring tool widely used to evaluate experiential avoidance and psychological rigidity (Karademas et al., 2017), which was translated into Chinese by Cao Jing and Zhang Chunqing in 2013 and 2014, and tested for reliability and validity (Cao et al., 2013). The Chinese version of the Acceptance and Action Questionnaire–II (AAQ-II) has “Adequate internal consistency reliability, test–retest reliability (one-month interval), factorial validity, and nomological validity with mindfulness, well-being, positive and negative affect/mood for both students and athletic samples.” The Chinese version of the AAQ-II may be a useful self-report measure of experiential avoidance in Chinese college students and Chinese athletes elite. AAQ-II is a 7-item single-dimensional questionnaire, adopting Likert’s 7-point scoring method, in which 1 means never and 7 means all correct. The test–retest reliability is 0.88, the higher the total score, the higher the degree of experiential avoidance and the lower the total score, the lower the degree of experiential avoidance (Zhang et al., 2014).

### 2.4 Self-reported questionnaire of non-intelligent factors for young athletes

The exercise anxiety subscale in the “Adolescent Athlete Non-Intellectual Factors Survey Scale” compiled by Chinese scholars Rong tunguo, Liu Yimin and others was used to measure the anxiety in the state of endurance exercise test. The subscale is composed of eight items, with test–retest reliability of 0.66 and a coefficient of 0.70 (Liu, 2003).

### 2.5 Exercise behavioral questionnaire and modified Borg CR10 RPE scale

The Exercise Negative Physical Sensations Questionnaire was assessed by utilizing the Modified Borg CR10 RPE scale. The Borg Rating of Perceived Exertion (RPE) scale was developed by Swedish researcher Gunnar Borg in the 1960s, and it is commonly referred to the Borg RPE scale. RPE serves as an outcome measure for determining exercise intensity prescription and plays a crucial role in monitoring progress and selecting appropriate modes of exercise during endurance training. The Modified Borg CR10 RPE scale survey was employed to investigate the adverse physical sensations experience during the pre-endurance exercise stress state. This scale is particularly suitable for assessing specific physical sensations, such as muscle pain or pulmonary responses (Williams, 2017). The reliability of Borg RPE in rating exertion was ascertained through testing, which was utilized in numerous studies (Lamb et al., 1999). Skinner et al. discovered no significant differences in physiological and perceptual variables related to work intensity when comparing the workload presented randomly with those obtained during the progressive exercise test (Skinner et al., 1973).

### 2.6 Sample size estimation

The minimum sample size in the mediation model simulation was determined based on three criteria: parameter bias, 95% coverage, and power. Additionally, the percentile Bootstrap method was employed to evaluate the sample size in the mediation model. Using Monte Carlo Power Analysis for Indirect Effects with a confidence interval of 95%, alpha = 0.05, power = 0.8, and 5,000 replications (Select Number of Monte Carlo Draws per Replications), we determined that a minimum sample size of 232 is necessary to achieve a power level of 0.80.^1^

According to Sim in a Monte Carlo simulation study to calculate the sample size, in the establishment of the Path Models, using the percentile Bootstrap method, under the assumption of partial intermediation, the Effect size is determined to be a medium amount of benefit (Effect size = 0.36) when the sample size required for 80, and the Effect size of the large and medium and small, the average of the sample sizes required for the three different Effect size levels was 227. For Structural Equation Models (SEM), the percentile Bootstrap method was used. The Effect size was determined as a moderate benefit (Effect size = 0.36) with a factor load of (Standardized factor loading) of 0.7. Assuming partial mediation, a sample size of 270 was deemed necessary. Similarly, Sim investigated 2,562 previous mediation model studies with a median sample size of 242 (Sim et al., 2022). Considering the potential for loss of follow-up, a sample size of 287 was determined for this study.

### 2.7 Data analysis

The data were collated and analyzed using SPSS 26.0 (IBM Corporation, New York, USA) statistical software. Tests for normality utilizing histograms and Q–Q plots indicated that the data exhibited conformity with a normal distribution. Descriptive statistics were employed to depict the demographics of the participants as well as the fundamental characteristics of endurance sport participation and statistics. Before regression analysis, it is assumed that all the independent variables and the dependent variable are the existence of a linear relationship, normal, independence, and equality of variance, and tested. Firstly, the linear relationship between the dependent and independent variables is initially demonstrated through a scatter plot. Subsequently, an analysis of the residual values using a histogram and goodness-of-fit test indicates their adherence to a normal distribution. The independence of error (residual) was verified by conducting a Durbin-Watson test (Durbin-Watson = 1.933). The Durbin-Watson statistic falls within the 0–4 range and is close to 2, indicating consistent data independence. The test for multicollinearity among variables, namely Experiential Avoidance (VIF = 1.114), Negative Physical Sensation (VIF = 1.310), and Endurance Exercise Behavior (VIF = 1.187), revealed that the VIF values for each variable were all below 5. Consequently, it can be inferred that there was no evidence of multicollinearity present. The histogram of residuals, in the meantime, exhibits a normal distribution with a mean close to 0 and a standard deviation close to 1 (representing a standard normal distribution), thereby indicating that the linearity assumption is met. The P–P plot further confirms the fulfillment of the normality condition. Consequently, this study satisfies the assumptions required for multiple linear regression.

Descriptive analyses were initially conducted, calculating and testing for significance of the Pearson correlations between the Endurance Exercise Behaviors score and the variables of Experiential Avoidance, Negative Physical Sensation and Exercise Anxiety in an exploratory analysis. Second, multiple regression analyses were performed. Three blocks of variables were entered as predictors in the analyses: (1) Endurance exercise behavior score; (2) negative physical sensation and (3) experiential avoidance questionnaire scales, which showed significant correlations with exercise anxiety in the preceding exploratory analysis (stepwise method). Based on the regression results and research hypotheses, we identified endurance exercise behavior scores as the independent variable, negative physical sensations and experiential avoidance as mediating variables, and exercise anxiety as the dependent variable. To examine the relationships between these factors, a validated factor analysis (CFA) was conducted using AMOS 24.0 (Amos Development Corporation). We reported multiple indexes of fit including chi-square, goodness-of-fit index (GFI), incremental-fit index (IFI), comparative-fit index (CFI), and root mean square error of approximation (RMSEA).

In order to enhance the precision of model parameter estimation, reduce variability caused by random sampling, improve stability, and minimize bias, a balanced approach was adopted in this study with 5,000 bootstrap iterations. This choice ensures both computational efficiency and time constraints while yielding scientific results. Consequently, we employed a bootstrap sampling method with 5,000 iterations to test the abovementioned mediation models. Statistical significance was determined at *p* < 0.05 level, and effect sizes for significant relationships were reported using eta square (*η*^2^) or partial eta square (*η_p_*^2^), along with their corresponding 95% confidence intervals (CI).

## 3 Results

### 3.1 Descriptive statistics and correlation analyses

A total of 287 freshmen and sophomores were coverage initiated, and the age distribution of the participants ranged from 17 to 24 years, with a mean age of 19.29, consisting of 192 females (66.9%) and 95 males (33.1%). The mean ± standard deviation of height for female students was 161.526 ± 5.110 cm (mean ± SD), weight was 56.849 ± 11.765 kg (mean ± SD), and the mean of BMI was 21.780 ± 4.423 (mean ± SD). The mean ± standard deviation of height for male students was 173.547 ± 5.079cn (mean ± SD), weight was 60.185 ± 13.144 kg (mean ± SD), and the mean of BMI was 21.919 ± 4.346. Exercise Anxiety of all participants was 17.2474 ± 5.8654 (mean ± SD). Experiential Avoidance was 17.648 ± 6.7568 (mean ± SD), Negative Physical Sensation was 7.0348 ± 0.83132 (mean ± SD), and Endurance Exercise Behavior was 5.069 ± 1.384 (mean ± SD). Using gender as the grouping variable, independent sample t-tests were performed on negative physical sensation, experiential avoidance and exercise anxiety. The results showed that there was no significant difference between males and females in the following three aspects (Table 1).

**Table 1:** Descriptive statistics and independent sample t-tests (*N* = 287).

	Woman		Man		t-test	
	Mean	SD	Mean	SD	*t*	*p*
Experiential avoidance	17.552	6.741	17.842	6.818	−0.340	0.734
Exercise anxiety	17.609	5.842	16.515	5.874	1.487	0.139
Negative physical sensation	7.094	0.8260	6.916	0.8336	1.707	0.089
Endurance exercise behavior	4.833	1.246	5.547	1.528	−3.950	<0.001

Pearson correlation analysis indicates that the negative physical sensation was found to be significantly positive associated with experiential avoidance (*r* = 0.308, *p* < 0.01, 95% Confidence Intervals, 95%CI = [0.200, 0.410]); The exercise anxiety was found to be significantly positive associated with negative physical sensation (*r* = 0.565, *p* < 0.01, 95%CI = [0.480, 0.639]) and experiential avoidance (*r* = 0.390, *p* < 0.01, 95%CI = [0.288, 0.484]); The endurance exercise was found to be significantly negatively associated with negative physical sensation (*r* = −0.409, *p* < 0.01, 95%CI = [−0.501, −0.308]), exercise anxiety (*r* = −0.375, *p* < 0.01, 95%CI = [−0.471, −0.271]) and experiential avoidance (*r* = −0.093, *p* < 0.01, 95%CI = [−0.207, 0.023]) (Table 2).

**Table 2:** Correlation analysis between variables (2-tailed, *N* = 287).

	M	SD	1	2	3	4
1. Negative physical sensation	7.035	0.8313	1	0.308^**^	0.565^**^	−0.409^**^
2. Experiential avoidance	17.648	6.757		1	0.390^**^	−0.093
3. Exercise anxiety	17.247	5.865			1	−0.375^**^
4. Endurance exercise behaviors	5.069	1.384				1

*p < 0.05, **p < 0.01. M, Mean; SD, Standard deviation.

### 3.2 Regression analyses

Taking exercise anxiety as the dependent variables, physical self-feeling, experiential avoidance and endurance exercise behavior as three independent variables, three multivariable regression equation models were established, respectively. Nonparametric percentile Bootstrap with deviation correction was used to test the model (Fang and Zhang, 2012). As shown in Table 3, when the three independent variables of negative physical sensations, experiential avoidance and endurance exercise behavior were successively included in the model, the *R* of the model reached the maximum of 0.619, and the *R*^2^ of the model reached the maximum of 0.398. The predictive effects of the three dependent variables on exercise anxiety all reached a significant level. Negative physical sensation (*β* = 0.414, *p* < 0.001, *η_p_*^2^ = 0.155) and experiential avoidance (*β* = 0.246, *p* < 0.001, *η_p_*^2^ = 0.336) showed a positive predictive effect on exercise anxiety, while endurance exercise behavior negatively predicted exercise anxiety (*β* = −0.183, *p* < 0.01, *η_p_*^2^ = 0.122).

**Table 3:** Hierarchical regression analysis examining negative physical sensation and experiential avoidance as mediators in the relationship between endurance exercise behaviors and exercise anxiety (*N* = 287).

Model item (model templates)		Model summary			Significant regression coefficient		95.0% confidence interval for B		Bootstraping^a^ BCa95% (Percentile confidence intervals)	
		*R*	*R*^2^	*F*	*β*	*t*	Lower	Upper	Lower	Upper
Model1	Negative physical sensation	0.565	0.319	133.443^***^	0.565	11.552^***^	3.306	4.663	3.243	4.669
Model2	Negative physical sensation	0.609	0.371	83.613^***^	0.491	9.921^***^	2.777	4.152	2.734	4.186
	Experiential avoidance				0.239	4.830^***^	0.123	0.292	0.113	0.296
Model3	Negative physical sensation	0.631	0.398	62.481^***^	0.428	8.112^***^	2.187	3.657	2.187	3.657
	Experiential avoidance				0.247	5.049^***^	0.13	0.296	0.13	0.296
	Endurance Exercise behaviors				−0.156	−3.088^**^	−1.196	−0.353	−1.196	−0.353

### 3.3 Examination of the proposed mediational model

**Figure 1:**
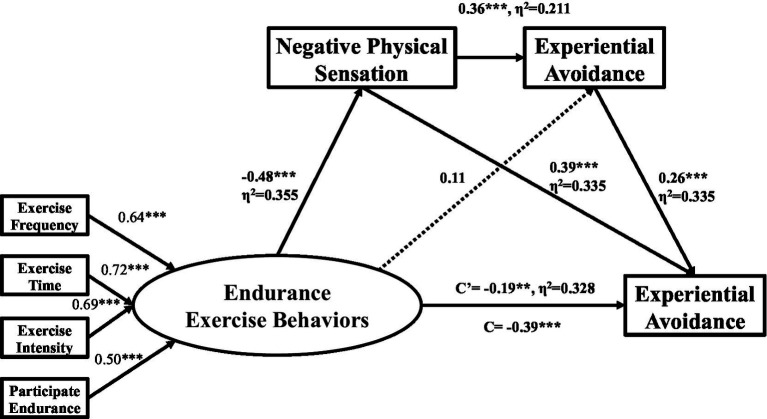
The chain-mediated model. Standardized regression coefficients for tests of serial indirect effects of endurance exercise behaviors on exercise anxiety via negative physical sensation and experiential avoidance (standardized *β*s). Path1 EEB → NPS → EA → EAN, path coefficient −0.044, *p* < 0.001, indirect effect = 11.19%. Path2 EEB → NPS → EAN, path coefficient −0.189, *p* < 0.001, indirect effect = 48.09%. Path3 EEB → EA → EAN, path coefficient 0.029, *p* = 0.160, indirect effect = 7.37%. Total indirect Effects = 51.98%. EEB, Endurance exercise behaviors; NPS, Negative physical sensation; EA, Experiential avoidance; EAN, Exercise anxiety; Dashed lines represent non-significant paths, C = Total Effects, C′ = Direct Effects; *p < 0.05, **p < 0.01, ***p < 0.001.

Taking exercise anxiety as dependent variable, endurance exercise behavior, negative physical experience and experiential avoidance as independent variables, the model was constructed in AMOS24. The results showed that the model fitted well: x2 = 28.484; df = 11, x2/df = 2.589; GFI = 0.972; AGFI = 0.929; NFI = 0.941; IFI = 0.963; TLI = 0.928; CFI = 0.962; RMSEA = 0.075, (Figure 1). Model fit was assessed by weighted least square mean (WLSM) and variance estimator, and compared using chi-square values, the comparative fit index (CFI), the Tucker-Lewis index (TLI), and the root-mean-square error of approximation (RMSEA). When the model satisfies the above indicators, it means that the model is well fitted, (RMSEA < 0.10, good model fit) (Breckler, 1990). x^2^/df < 2, the model is a very good fit. x^2^/df values greater than 2 and less than 5 are usually acceptable, (x^2^/df < 3, good model fit) (Marsh et al., 1988). Lower RMSEA values indicate better model fit (<0.10 = good model fit) (Steiger, 1990). Higher CFI and TLI values indicate better model fit (>0.95 = good model fit) (Breckler, 1990).

Figure 1 presents the path coefficients from the bootstrapped regression and mediation analyses for the effects of endurance exercise behaviors on exercise anxiety through a sequential path of negative physical sensation followed by experiential avoidance. The overall regression model examining the relationship between endurance exercise behaviors and exercise anxiety was statistically significant (*R*^2^ = 0.384, *F*_1,283_ = 58.70, *p* < 0.001). Specifically, endurance exercise behaviors significantly and negatively predicted negative physical sensation (*β* = −0.48, *p* < 0.001, *η*^2^ = 0.355, 95% confidence interval (CI): [0.109, 0.305]). Endurance exercise behavior will reduce the individual’s negative physical sensation. Negative physical sensation significantly and positively predicted experiential avoidance (*β* = 0.36, *p* < 0.001, *η*^2^ = 0.211, 95% confidence interval (CI): [0.000, 0.126]). As the negative physical sensation increases, so does individual avoidance increases. Negative physical sensation and experiential avoidance significantly positively predicted exercise anxiety (*β* = 0.39, *p* < 0.001, *η*^2^ = 0.335, 95% confidence interval (CI): [0.242, 0.405]; *β* = 0.26, *p* < 0.001, *η*^2^ = 0.253, 95% confidence interval (CI): [0.086, 0.259]). Therefore, the increase in negative physical sensation and experiential avoidance will increase exercise anxiety. In this model, the direct effect of endurance exercise behaviors on exercise anxiety was statistically significant (*β* = −0.19, *p* < 0.001, *η*^2^ = 0.328, 95% confidence interval (CI): [0.079, 0.275]). Therefore, there is a partial mediating effect between endurance exercise behavior and exercise anxiety.

Three indirect effect paths were established, respectively. Path 1: Endurance exercise behavior → Negative physical sensations → Experiential avoidance → Exercise anxiety. Path 2: Endurance exercise behavior → Negative physical sensations → Exercise anxiety. Path 3: Endurance exercise behavior → Experiential avoidance → Exercise anxiety. The Bootstrap percentile method was used to test the statistical significance of the mediation effects. The Perform bootstrap sample size was 5,000 (Number of bootstrap samples), and the percentile position confidence interval was 95% (PC confidence level). The results are shown in Table 4.

**Table 4:** Hierarchical regression analysis examining negative physical sensation and experiential avoidance as mediators in the relationship between endurance exercise behaviors and exercise anxiety (*N* = 287).

Path		*β*	Boot Mean	Boot SE	*Z*	Bias-Corrected 95%CI			Effect	Status
						Lower	Upper	p		
Path1	EEB → NPS → EA → EAN	−0.044	−0.045	0.015	−3.041**	−0.081	−0.022	0.000	11.19%	Supported
Path2	EEB → NPS → EAN	−0.189	−0.188	0.037	−5.142***	−0.269	−0.123	0.000	48.09%	Supported
Path3	EEB → EA → EAN	0.029	0.029	0.023	1.260	−0.010	0.077	0.160	7.37%	Not supported
	Total indirect effects	−0.204	−0.203	0.044	−4.637***	−0.297	−0.124	0.000	51.98%	Supported

EEB, Endurance exercise behaviors; NPS, Negative physical sensation; EA, Experiential avoidance; EAN, Exercise anxiety; *p < 0.05, **p < 0.01, ***p < 0.001.

The standard indirect effect value of path 1 was *β* = −0.044, *p* < 0.01, accounting for 11.19% of the total indirect effect. Bootstrap 95% confidence interval did not contain 0, indicating that the mediating effect was significant. The standard indirect effect value of path 2 was *β* = −0.189, *p* < 0.001, accounting for 48.09% of the total effect. Bootstrap 95% confidence interval did not contain 0, indicating that the mediating effect was significant. The standard indirect effect value of path 3 of endurance exercise behavior → experiential avoidance → exercise anxiety was *β* = −0.029, *p* > 0.05, accounting for 7.37% of the total indirect effect, and the bootstrap 95% confidence interval of path 3 included 0, indicating that the mediating effect of this path was not significant.

Therefore, the negative physical sensations and experiential avoidance play a partial mediating effect in the influence of endurance exercise behavior on exercise anxiety. The standard mediation effect value is −0.204, and the explanation of the total mediation effect is 51.98%.

## 4 Discussion

The correlation analyses revealed a significant inverse relationship between endurance exercise behavior and negative physical sensations, as well as experiential avoidance in endurance sports. Additionally, a significant positive association was observed between negative physical sensations, experiential avoidance, and exercise anxiety. Regression analyses revealed a negative association between endurance exercise behavior and negative physical sensations as well as experiential avoidance. It was found that both negative body sensations and experiential avoidance positively predicted exercise anxiety. Therefore, the model suggests that negative physical sensations and experiential avoidance play a chain-mediated moderating role in predicting exercise anxiety associated with endurance behaviors. The findings were further supported by employing structural equation modeling, which revealed that individuals engaging in exercise and having a predisposition to anxiety effectively regulate exercise-induced anxiety through a pathway involving negative physical sensations leading to experiential avoidance.

Athletic anxiety is currently acknowledged as a manifestation of state anxiety within specific environmental contexts (Walter et al., 2019). Spielberger proposed the State–State Anxiety Theory (SSAT), which posits that state anxiety is an immediate and modifiable ‘emotional state’ characterized by a distinct amalgamation of tension, apprehension, nervousness, intrusive thoughts and concerns, and physiological alterations. Adverse influence in competitive anxiety states were more substantial within the physical dimension than within the cognitive dimension (Ries et al., 2012). The present study further demonstrates that this physical anxiety, which exerts a more detrimental impact compared to cognitive anxiety, is also prevalent among the general college student population through the examination of chain-mediated effects.

There is a popular belief that the lack of willpower/self-discipline is widely acknowledged as a significant barrier for individuals who do not engage in physical activity/exercise. Moreover, individuals with anxiety and depression often struggle with motivation and consistency when it comes to physical activities that require effort. To promote participation in physical activity, it is essential to establish a favorable social environment, cultivate positive beliefs about the benefits of exercise, provide well-equipped sports facilities, and offer professional sports instruction (Crichton et al., 2024). Individuals with depression may exhibit reduced engagement in physical activity due to diminished interest and their energy levels. Similarly, individuals with anxiety may avoid exercising in public settings due to social fears. Additionally, negative factors such as poor self-perceived physical and mental health, low self-efficacy, and limited self-confidence can impede their participation in physical activities (Horenstein et al., 2021). These factors collectively contribute to a detrimental cycle of negative feedback, exacerbating the state of mental health (Marashi et al., 2021). In return, mental disorders such as anxiety and depression can exacerbate physical health risks by influencing health behaviors. Consequently, the reciprocal feedback loop between physical and mental states is disrupted, leading to altered perception and evaluation of physical sensations. This bidirectional relationship between mental disorders and physical health establishes a self-perpetuating feedback loop that contributes to elevated levels of both mental disorders and physical health ailments (Scott et al., 2009). The adverse association is additionally substantiated by the finding that exercise anxiety in the current study’s model is mediated by negative physical sensations and experiential avoidance.

The Psychobiological Model posits that any factor which diminishes the perception of effort or enhances latent motivation is conducive to improving endurance performance, whereas any factor that heightens the perception of effort or dampens latent motivation hampers endurance performance (Marcora et al., 2008). Endurance sports involve the continuous and dynamic repetition of movements using whole-body strength over an extended period. As such, athletes must maintain motivation and perseverance despite challenges such as pain or injury (McCormick and Hatzigeorgiadis, 2019). Research has demonstrated a positive correlation between perception of effort and pain in endurance sports (Astokorki and Mauger, 2017). Consequently, physical sensations like pain can heighten an individual’s perception of effort during endurance exercise, ultimately impairing performance. Mauger (2014) contends that exercise-induced muscle discomfort may also impact pacing decisions and overall endurance performance, particularly during high-intensity activities (Kress and Statler, 2007). Performers feels increasingly unpleasant between the lactate and ventilatory thresholds and maximal oxygen uptake in endurance exercise (Ekkekakis et al., 2011), and their unpleasant feeling could influence pacing decisions, particularly in populations that are inexperienced or non-competitive. Consequently, this adverse affective response may concurrently heighten an individual’s perception of effort, pain, and affective valence, thereby compromising endurance sport performance (McCormick and Hatzigeorgiadis, 2019).

Negative bodily experiences can divert an individual’s attention away from the intended task and toward physical sensations, potentially triggering avoidant safety behaviors (Mercader-Rubio et al., 2023). Numerous stressors, including negative physical sensations, elicit assessments of stress from athletes. The consequences of these adverse evaluations can lead endurance athletes to experience detrimental emotional responses such as anxiety, frustration, and discouragement before and during competition, which in turn negatively impact their motivation and attention (McCormick et al., 2016). The stress induced by various unfavorable physical sensations alters the context within which pre-existing cognitive strategies like perception of effort, pain, and emotional valence operate. Under the strong influence of multiple negative emotions, individuals then employ an emotion regulation strategy primarily centered around inhibition. Such inhibitory response regulation strategies predominantly involve avoidance behaviors, suppression of negative emotional experiences, and cognitive control aimed at modifying attentional focus. It further proves that the evaluation of emotions as negative or rejection-oriented mediates the intensity of negative emotion and emotional inhibition (Laura et al., 2006). An individual’s assessment and response to emotions triggered by external circumstances or physical symptoms will determine subsequent coping strategies across three domains: cognitive, affective, and behavioral. If the “meta emotion” of this initial judgment is negative, it will start avoidance coping strategies. Therefore, the emotional judgment and cognitive strategies of physical symptoms before and during endurance exercise are the key factors affecting endurance exercise behavior and exercise anxiety, which is well proved by the model relationship in the study. Findings suggest that negative feelings about the body and experiential avoidance may be overall risk factors for later symptoms of exercise anxiety in college students relative to a diagnosis of exercise anxiety.

Although these inhibitory conditioning strategies offer temporary relief from negative emotions, the paradoxical effect of inhibition reinforces the pursuit of inhibition and resurfaces, diverting attention from the intended goal, intensifying individual distress, amplifying perceived effort, and diminishing self-efficacy. Consequently, this may undermine endurance performance and even result in disruption and disengagement from endurance practice (Borrajo et al., 2023). Therefore, anxiety represents a form of stress response to a perceived aversive situation that triggers avoidance mechanisms and is characterized by apprehension and fear regarding potential physical or psychological harm, accompanied by heightened physiological arousal due to threat assessment (Pineda-Espejel et al., 2019). The physiological activation subsequently intensifies negative emotional reactions, concerns, and thoughts, thereby influencing attentional processes and other cognitive functions, emotional experiences, and behaviors (Pulido et al., 2021), falling into a negative cycle. Moreover, the frequent utilization of temporary inhibitory strategies, encompassing cognitive, behavioral, and emotional aspects poses a heightened risk of automating and rigidifying avoidance mechanisms, consequently leading to experiential avoidance. Forsyth posits that anxiety disorders are significantly influenced by avoidance behaviors, including experiential avoidance (Olatunji et al., 2007).

Hayes argues that experiential avoidance leads to the consolidation of negative experiences, activation of negative responses, and a shift toward employing avoidance strategies, thereby diminishing an individual’s engagement in new behaviors and impeding the exploration of novel cognitive and emotional experiences (Hayes et al., 2006). At the same time, it is unnecessary to mobilize a large number of cognitive resources to temporarily restrain, distort and cover negative experience, which only consumes cognitive resources, weakens value objectives, affects individual behavior and causes functional loss (Hayes et al., 2004). Therefore, the negative physical sensation and experiential avoidance increase anxiety, and play an important role in the process of transforming anxiety from ordinary cognitive activities to pathological mental disorders (Akbari and Khanipour, 2018). Consequently, this negative evaluation of physical sensations is closely linked to experiential avoidance, which in turn manifests as behavioral expressions of experiential avoidance within the context of anxiety and is associated with detrimental health outcomes. For instance, inhibiting emotions exacerbates subjective distress and physiological disorders, resulting in compromised physical well-being. Therefore, experiential avoidance can be considered a general risk factor for physical health issues in individuals with anxiety disorders and plays an alternative role in influencing their physical health (Berghoff et al., 2017). The findings from our study further corroborate this perspective by emphasizing the contribution of experiential avoidance as a potential factor underlying the heightened manifestation of physical health symptoms among individuals with anxiety disorder. The anxiety model of endurance exercise established in this research just proves this theory. Negative physical sensation and experiential avoidance positively predict exercise anxiety. The participation of endurance exercise behaviors reversely predicts negative physical sensation, experiential avoidance and exercise anxiety; participation in endurance exercise can reduce the negative physical sensation, and then reduce experiential avoidance and exercise anxiety. This is also consistent with the results of Hossein et al. that endurance exercise can reduce anxiety levels in rats through animal models (TaheriChadorneshin et al., 2017). Experiential avoidance can be considered a general risk factor for physical health issues in individuals with anxiety disorders and plays an alternative role in influencing their physical health.

This study revealed the relationship model among endurance exercise behavior, negative physical sensation, experiential avoidance and exercise anxiety. The negative physical sensation and experiential avoidance in endurance exercise are the key factors affecting anxiety. Negative physical sensations and experiential avoidance are influential factors in regulating exercise anxiety during endurance activities, with a chain mediating role played by negative physical sensations and experiential avoidance in the context of exercise anxiety during endurance exercise behavior. Therefore, interventions targeting anxiety in endurance exercise can facilitate the development of new physical cognition and emotional experiences through active participation in endurance exercise. By enhancing pleasurable and rewarding activation between participants and exercise environment, negative solidified cognitions and avoidance behaviors toward endurance exercise can be eliminated. Consequently, positive physical sensations and emotional experiences can be promoted through engagement in endurance exercise behavior, ultimately reducing exercise anxiety (Ekers et al., 2014).

## 5 Limitations and future perspectives

The present study employed a cross-sectional design, utilizing questionnaire-based measurement methods exclusively for data collection through self-reporting. This study method limits causal inference and introduces quite a few limitations to inference. At the same time, the study design did not allow for the stability of the problem assessment over time. Therefore, no longitudinal studies were identified that consistently tracked physical activity levels of the participants, which would have minimized the risk of misclassification bias. In future investigations, we will integrate physician-provided medical diagnostics with wearable devices capable of capturing physiological indicators such as EEG, skin conductance, heart rate variability, and blood pressure in order to objectively assess the relationships elucidated in this article. Another limitations lies in our cautious interpretation of the results due to the relatively small sample size. Therefore, conducting a subsequent study with a larger sample size would be beneficial for validating these findings.

Furthermore, this study examined physical sensation and exercise anxiety in a nonclinical college sample characterized by a relatively low average level of reported symptoms. Consequently, the generalizability of our findings to individuals with more severe physical health conditions may be limited. Therefore incorporating diverse groups, such as professional athletes or patients, in future investigations could elucidate the relationship between experiential avoidance and anxiety disorders with significant physical health conditions. In addition, future studies would benefit from corroborating self-reported physical activity exposures with device-based assessments, and in selecting the physical activity measure, the physical activity dose advocated by the International Health Organization is recommended for ease of generalization and comparison.

The intensity of physical activity plays a crucial role in anxiety interventions, and current research suggests that light physical activity is more practical and accessible. However, the relationship between the intensity and dosage of physical activity and avoidance behaviors as well as exercise-related anxiety remains unclear. If the association between physical activity and anxiety indeed varies according to physical activity dose, the observed heterogeneity in doses across studies may partially account for this inconsistency. Determining whether these associations are dose-dependent is crucial not only for guiding recommendations in clinical practice and public health policy but also for meeting evidentiary criteria supporting a causal relationship between exposure to physical activity and anxiety outcomes (McDowell et al., 2019). This aspect warrants further investigation in future research.

Indeed, the likely bidirectional nature of the relation between physical health problems and anxiety complicates efforts to pinpoint the development of each, as well as to disentangle the processes that link anxiety diagnoses and physical health problems (Duric et al., 2016). Potential reciprocal relations between anxiety pathology and physical health symptoms remain an important area for future research (Berghoff et al., 2017).

## 6 Conclusion

There are many significant correlations among endurance exercise behavior, negative physical sensations, experiential avoidance and exercise anxiety; endurance exercise behavior affects exercise anxiety through two ways: endurance exercise → negative physical sensations → experiential avoidance → exercise anxiety; endurance exercise → negative physical sensations → exercise anxiety. Negative physical sensation and experiential avoidance play a mediating and chain mediating role between endurance exercise behavior and exercise anxiety. Therefore, endurance exercise behavior can reduce exercise anxiety by reducing negative physical sensations and experiential avoidance. Therefore, for those who are sedentary and worried about participating in endurance sports exercise, it is crucial to mitigate their adverse physical sensations during such activities. By doing so, we can effectively alleviate negative emotions and avoidance tendencies toward endurance exercise, thereby reducing exercise anxiety and fostering increased participation in this domain.

## Data Availability

The raw data supporting the conclusions of this article will be made available by the authors without undue reservation.

## Footnotes

^1^https://schoemanna.shinyapps.io/mc_power_med/
