# Supplementary material for: The chain-mediating effects of negative physical sensation and experiential avoidance on exercise anxiety in college students
Source: Front Psychol. 2024 Nov 26;15:1465424. doi: 10.3389/fpsyg.2024.1465424 (PMC11629875; doi:10.3389/fpsyg.2024.1465424)
Supplement: Supplementary file 1 [file Table_1.DOC]

Multiple linear regression analysis hypothesis condition test£º


A scatter plot matrix between the respective variable (endurance exercise behavior scores, negative body sensations, experiential avoidance) and the dependent variable (exercise anxiety)


Model Summaryb	
Model	R	R Square	Adjusted R Square	Std. Error of the Estimate	Durbin-Watson	
1	.619a	.384	.377	4.62941	1.933	

a. Predictors: (Constant), Endurance Exercise Behavior, Experiential Avoidance, Negative Physical Sensation	
b. Dependent Variable: Exercise Anxiety	


ANOVAa	
Model	Sum of Squares	df	Mean Square	F	Sig.	
1	Regression	3774.348	3	1258.116	58.704	.000b	
	Residual	6065.088	283	21.431			
	Total	9839.436	286				

a. Dependent Variable: Exercise Anxiety	
b. Predictors: (Constant), Endurance Exercise Behavior, Experiential Avoidance, Negative Physical Sensation	


Coefficientsa	
Model	Unstandardized Coefficients	Standardized Coefficients	t	Sig.	Collinearity Statistics	
	B	Std. Error	Beta			Tolerance	VIF	
1	(Constant)	-7.745	2.644		-2.929	.004			
	Experiential Avoidance	.217	.043	.250	5.074	.000	.897	1.114	
	Negative Physical Sensation	3.101	.377	.439	8.229	.000	.764	1.310	
	Endurance Exercise Behavior	-.008	.003	-.124	-2.442	.015	.843	1.187	
a. Dependent Variable: Exercise Anxiety	


Residuals Statisticsa	
	Minimum	Maximum	Mean	Std. Deviation	N	
Predicted Value	9.5810	31.6867	17.2474	3.63277	287	
Residual	-10.36638	17.20429	.00000	4.60506	287	
Std. Predicted Value	-2.110	3.975	.000	1.000	287	
Std. Residual	-2.239	3.716	.000	.995	287	

a. Dependent Variable: Exercise Anxiety	

harts£º


Normality test for the dependent variable£º

Tests of Normality	
	Kolmogorov-Smirnova	Shapiro-Wilk	
	Statistic	df	Sig.	Statistic	df	Sig.	
Exercise Anxiety	.069	287	.002	.989	287	.027	

a. Lilliefors Significance Correction	
